# Educational technology to empower patients as participants in their care

**DOI:** 10.1590/0034-7167-2023-0359

**Published:** 2024-12-16

**Authors:** Rodrigo da Silva Ramos, Mateus Ferreira de Aguiar, Eurinete Catarina Guimarães da Silva, Cleberson Morais Caetano, Rizioléia Marina Pinheiro Pina, Joice Claret Neves, Hadelândia Milon de Oliveira

**Affiliations:** I Secretaria Municipal de Saúde de Manaus. Manaus, Amazonas, Brazil; II Universidade Estadual do Amazonas. Manaus, Amazonas, Brazil; III Universidade Federal do Amazonas. Manaus, Amazonas, Brazil; IV Empresa Brazileira de Serviço Hospitalares. Florianópolis, Santa Catarina, Brazil

**Keywords:** Patient Safety, Health Education, Educational Technology, Patients, Nursing., Seguridad del Paciente, Educación Sanitaria, Tecnología Educativa, Pacientes, Enfermería.

## Abstract

**Objectives:**

to build and validate an educational technology to empower patients as participants in their own care.

**Methods:**

methodological study to develop an educational technology based on the elaboration, validation, and evaluation that were carried out in five stages at a teaching hospital in Amazonas. The study was carried out from 2019 to 2022, with the participation of 19 judges specialized in patient safety and 72 patients admitted to the hospital’s medical and surgical clinics, the study setting.

**Results:**

the agreement between the judges obtained an overall index of 0.85, being considered validated. The overall analysis of the booklet obtained an assessment of the level of agreement above 85%.

**Final Considerations:**

the educational technology presented here was validated and suitable for promoting patient/professional rapprochement and consolidating health care in a way that increases the patient’s ability to contribute to their treatment and prevent the occurrence of adverse events.

## RESUMEN

**Objetivos:**

construir y validar una tecnología educativa para empoderar al paciente como partícipe de su cuidado.

**Métodos:**

es un estudio metodológico llevado a cabo a partir de la elaboración, validación y evaluación de una tecnología educativa, realizado en cinco etapas en un Hospital escuela del Amazonas. El estudio transcurrió durante el período de 2019 a 2022, con la participación de19 jueces especialistas sobre seguridad del paciente y 72 personas internadas en las clínicas médicas y quirúrgicas del hospital, escenario del estudio.

**Resultados:**

el acuerdo entre los jueces obtuvo un índice global de 0,85, considerado como validado. El nivel de acuerdo del análisis global del folleto se estimó superior al 85%.

**Consideraciones Finales:**

la tecnología educativa aquí presentada fue validada y es apta para promover la aproximación paciente/profesional y consolidar la atención sanitaria de tal manera que aumente la capacidad del paciente en beneficio de su tratamiento, evitando así, eventos adversos.

## INTRODUCTION

Patient safety has been broadened in its concept, defined as a structure of organized activities that creates cultures, processes, procedures, behaviors, technologies and environments in the health area with lower risks in a consistent and sustainable manner, to mitigate the occurrence of preventable harm and reduce its impacts when they occur^(1)^.

It is estimated that, in low-income countries, one in four patients is exposed to an incident with harm in health services^(2)^. On average, 60% of deaths occur due to unsafe practices and poor quality of care provided^(3)^. Adverse events are the result of systemic or organizational errors that favor the occurrence of failure in the care provided^(4)^.

However, if quality assurance in health is only through results, senior management will centralize efforts in processes, actions and products, which may depreciate relationships and individual patient preferences. Therefore, rethinking health care as co-production can bring benefits to improving the quality of health services^(5)^.

The 2021-2030 Global Patient Safety Action Plan, which seeks to eliminate preventable harm in healthcare, has seven guiding principles to guide actions, with two of these principles focusing on the patient in favor of harm-free healthcare, namely: engaging patients and families as partners in safe care and using the patient experience to improve safety^(6)^.

In this context, knowing the perspective of patients and their families has been a priority, including to help build patient-centered care processes and improve the performance of clinical teams^(7)^. Therefore, the healthcare team must accurately guide patients, using educational materials that facilitate their understanding.

Patients and caregivers, properly guided and empowered by self-care, are able to report incidents and contributing factors without prejudice, providing new and valuable information about the type and frequency of these occurrences, which enables the improvement of the quality of care, with a basis for shared and assertive decision-making^(8)^.

The use of printed educational technologies, such as flipcharts and booklets, is a viable alternative for informing and raising awareness among patients, for health promotion, in addition to allowing for later reading, which reinforces verbal instructions, serving as a guide in cases of doubts^(9)^.

The development, validation and implementation of educational materials in the form of booklets has been repeated in national and international studies, and it is important that educational tools on patient safety are developed and validated for use in clinical practice with the aim of contributing to the dissemination of the culture of patient safety in health services^(10)^. The booklet is a mechanism considered to have greater financial viability in the dissemination of information^(11)^.

Therefore, it is important to discuss teaching methodologies that are capable of assisting patients and family members in their self-care, facilitating the understanding of the pillars of patient safety.

The educational booklet is a potential ally in clarifying ideas and as a source of information for patients. For patient safety, this technology has the capacity to guide patients regarding hospital admissions and guide them in their engagement in safe care^(12)^.

In view of the above, the importance of this study for the construction, validation and evaluation of an Educational Technology is highlighted, as it contemplates three axes of Patient Safety, namely: it stimulates safe care practices, citizen involvement in safety and promotes research on patient safety^(13)^.

## OBJETICVES

To build and validate an educational technology to empower patients as participants in their own safe care.

## METHODS

### Ethical aspects

The research was approved by the Research Ethics Committee of the Federal University of Amazonas (UFAM), through Brazil Platform, in accordance with Resolution 466/2012 (CNS, 2012). Both in the validation phases, carried out by expert judges, and in the evaluation phases, carried out by patients, all were informed about the objectives of the study and agreed to participate through the Free and Informed Consent Form (FICF), signed in two copies, one with each participant and the other with the research team.

### Theoretical-methodological framework

The construction of the educational booklet (EB) was guided by the concepts of the Andragogical model, which, because it is flexible, may or may not be adopted in its entirety, since the strength of Andragogy “lies in a set of six fundamental principles on Adult Learning that apply to all learning situations”^(14)^.

Its premises are: 1. The need to know: the adult needs to know why he or she should learn something before actually starting to learn; 2. The learner’s self-concept: the adult sees himself or herself as responsible for his or her own life and decisions, and wants to be seen and treated by others as capable of self-direction; 3. The role of experience: the adult accumulates a set of experiences that become an inexhaustible source of learning; 4. Readiness to learn: the adult engages in learning that which can help him or her solve real-life problems and perform his or her social roles; 5. Orientation to learning: the adult’s temporal perspective is focused on the immediate application of knowledge; 6. Motivation: extrinsic factors and intrinsic factors^(14)^.

In the context of patient safety, where the adult is hospitalized, it is understood that Andragogy is capable of designating fundamental principles to contribute to the teaching-learning process in a critical and reflective way, to strengthen learning and empower the patient as a participant in their care.

### Type of study

This is a methodological study to construct a validation EB, with judges and evaluation with the target audience, guided by the Consolidated Criteria for Reporting Qualitative Research (COREQ) tool, made available by the EQUATOR Network, which guides the description of qualitative studies^(15)^.

### Methodological procedures

The study was conducted in five stages: identification of the knowledge of users of a teaching hospital about the care that contributes to patient safety; literature review; construction of the CB; validation of the content and appearance of the CB by the judges; evaluation of the content and appearance of the CB by the target audience^(16)^.

In stage 1, a descriptive-qualitative study was conducted to identify the knowledge of users of a teaching hospital about the care that contributes to patient safety^(17)^, relevant to understanding the knowledge of the research subject about patient safety in the hospital environment to guide the construction of the CB.

In stage 2, a bibliographic search was conducted, registered on the PROSPERO platform and developed using the Scoping Review method. The guiding question was constructed using the mnemonic combination PCC as a strategy: P Population - adult patients; C Concept - educational technologies in health; C Context - patient safety. Thus, the following research question was defined: what and how are educational technologies in health being applied to patients in the context of their own safety?

The research was carried out in the databases via journals of the Coordination for the Improvement of Higher Education Personnel (Capes): Virtual Health Library (BVS), Latin American and Caribbean Literature in Health Sciences (LILACS), Medical Literature Analysis and Retrieval System Online (MEDLINE) and Scientific Electronic Library Online (SCIELO), Public/Publisher MEDLINE (PubMed), SciVerse SCOPUS and Nursing Database (BDENF). The research included articles in Portuguese, Spanish and English, published between 2010 and 2020, which were available for reading in full.

In stage 3, the booklet was prepared, carried out from May to August 2020, considering the statements of the patients interviewed and a bibliographic survey of recommendations on patient safety from international bodies (World Health Organization and Pan American Health Organization), Ministry of Health Manuals and the results of the scoping review.

The content, illustration and layout of the educational technology were developed together with the researchers involved in the study and a graphic programming/design professional, observing the criteria related to content, structure, organization, language, layout, design, cultural sensitivity and suitability for patients in hospitalization, with prior presentation to the nurses who are members of the Patient Safety Center of the University Hospital, where the booklet was later applied.

In stage 4, the educational technology was validated using the Content Validity Index by expert judges, from February to August 2020. During the initial contact, the expert judges were invited to participate in the research, containing the instructions and the Free and Informed Consent Form (FICF). After acceptance, the expert judges received the Likert Scale and the educational booklet.

In stage 5, the content and appearance of the educational booklet were evaluated by the target audience, applied individually to each patient. After signing the FICF, each patient received a copy of the patient safety educational booklet. After reading, the assessment instrument was given to the patients, who completed it within 15 to 20 minutes. Data collection took place between May and June 2022.

### Study environment

The study was carried out in a teaching hospital linked to the Federal University, a reference in medium and high complexity care throughout the Western Amazon, also working in the training of undergraduate and postgraduate health professionals and the development of scientific research and university extension.

### Data Source

For content validation, 20 expert judges were invited, of which 19 agreed to participate in the survey. The following eligibility criteria were used: a) having at least 3 years of experience in patient safety and/or care for inpatient clinical or surgical patients; b) having at least one lato sensu specialization in health. The survey of eligible expert judges was conducted through the Lattes Platform of the National Council for Scientific and Technological Development (CNPq) portal, using the following filters: patient safety and specialization in the subject. Only one was excluded because he answered the questionnaire more than 30 days ago.

Thirty-six patients participated in the evaluation of content and appearance by the target audience. The selection criteria used were: patients admitted for the first time to the clinical medical and surgical units, regardless of age, sex and medical diagnosis, with a minimum stay of 24 hours. Patients with reduced level of consciousness, clinically unstable, and patients scheduled for internal or external transfer to other clinical units during the data collection period were excluded. The target audience was surveyed directly in the inpatient units, by prior survey of the patient’s clinical data in the medical records, and then the eligible patient was approached directly for the study. The inclusion of inpatients is justified, since these patients experience safe care with a lower risk of failures in the care provided during their hospital stay, and their involvement in care is essential.

### Data collection and organization

In stage 4 of the validation of the educational technology using the Content Validity Index by expert judges, the instrument used has three parts: identification, instructions and blocks of questions with the following structure: a) objectives; b) structure and presentation; c) relevance; d) general comments and suggestions. The Likert Scale consisted of the following items: Totally Adequate (TA); Adequate (A); Partially Adequate (PA); Inadequate (I).

The deadline for feedback by the judges was up to 15 days. Those who did not return the scale within the proposed periods had an additional 7 days to send it and, finally, those who did not send it were excluded from the research.

In stage 5, the content and appearance of the educational booklet were evaluated by the target audience, using an Instrument containing 6 items: 1. Literary Presentation; 2. Illustrations; 3. Sufficiently expressive and comprehensive material; 4. Legibility and printing characteristics; 5. Quality of Information; 6. Personal opinions (the latter with a descriptive aspect). With answers for each item being: “Yes”, “No” or “Partially”.

### Data analysis

For content validation, the Content Validity Index (CVI) score was calculated, which allows each item to be analyzed separately and as a whole. The statistical analysis used the percentage of valid responses for each item, and validation was performed by block, that is, the average response for each item.

In the validation by the judges, the sums of the responses for the items “Totally Adequate” and “Adequate” were used. After this calculation, the minimum expected CVI was greater than or equal to 0.70 or 70% approval. The agreement between the judges obtained an overall CVI of 0.85 in the first evaluation, and it was considered validated.

The suggestions of the expert judges regarding the first version of the booklet were met with regard to: changing repeated phrases, correcting Portuguese errors, changing page numbers, changing texts for better understanding and adding new suggested texts (in the items Falls, Safe Medication Administration, Safe Surgery and Hand Hygiene).

The target audience’s assessment was carried out using the responses to each item, which were “Yes”, “No” or “Partially”. Items with a “Yes” or “Partially” response were considered valid. Items with a minimum agreement rate of 75% were considered validated. Items with a lower agreement rate were considered subject to change.

## RESULTS

In the first stage of the descriptive-qualitative study, to identify the knowledge of users of a teaching hospital about the care that contributes to patient safety, 72 patients were interviewed, 52 (72.2%) of whom were female and 20 (27.8%) were male, with the highest participation rate being between 41 and 46 years old. Regarding the hospitalization clinic, 6 (8.3%) were hospitalized in the Medical Clinic and 66 (91.7%) in the Surgical Clinic of the University Hospital studied. Three categories emerged from Bardin’s analysis: “Patient Care and Safety”; “Dialogue with the team”; “Confirmation of procedures and trust”^(17)^. The literature review identified different types and methods of Educational Technologies applied to patients and family members to help them engage in their safe care, which can come in a variety of forms - from the simplest and most traditional to digital ones with greater technological resources for disseminating knowledge remotely, which are efficient in the context of the novel Coronavirus pandemic.

The technology was validated by 19 expert judges, the majority of whom were female (76.7%). Regarding professional training, 89.7% were nurses and 10.3% pharmacists. Regarding qualifications, 51.7% had doctorates, 10.3% had master’s degrees and 38% were specialists.

Thirty-six patients admitted to medical (32%) and surgical (68%) clinics participated in the interviews for the assessment stage of the booklet by the target audience, all of whom were first-time hospitalized, regardless of gender, diagnosis and with a minimum hospital stay of 24 hours.

The agreement between the judges obtained an overall CVI of 0.85 in the first assessment, and was considered validated. The sums of the responses for the items Totally Adequate and Adequate were used. Table 1 presents the individual and global indexes for each item evaluated.

**Table 1:** Assessment of the adequacy of the educational booklet regarding the Content Validity Index, Manaus, Amazonas, Brazil, 2022

Domain / Assessment Items	CVI N19(%)
1. Objective (CVI)	0.86%
1.1 The information/content is important for the quality of work/quality of life of the TE target audience	0.96%
1.2 It invites and/or instigates changes in behavior and attitude	0.89%
1.3 It can circulate in the scientific community of the area	0.82%
1.4 It meets the objectives of institutions where the ET target audience works/serves	0.75%
2. Structure and Presentation (CVI)	0.83%
2.1 The ET is appropriate for the audience	0.86%
2.2 The messages are presented in a clear and objective manner	0.68%
2.3 The information presented is scientifically correct	0.93%
2.4 The material is appropriate to the sociocultural level of the ET's target audi-ence	0.78%
2.5 There is a logical sequence of the proposed content	0.93%
2.6 The information is well structured in terms of agreement and spelling	0.79%
2.7 The writing style corresponds to the target audience's level of knowledge	0.78%
2.8 The information on the cover, back cover, summary, acknowledgments and/or presentation is coherent	0.80%
2.9 The size of the title and topics is appropriate	0.72%
2.10 The illustrations are expressive and sufficient	0.96%
2.11 The material (paper/printing) is appropriate	NA
2.12 The number of pages is appropriate	0.89%
3. Relevance (CVI)	0.88%
3.1 The themes portray key aspects that should be reinforced	1.00%
3.2 TE allows the transfer and generalization of learning to different contexts	0.78%
3.3 TE proposes the construction of knowledge	0.96%
3.4 TE addresses the subjects necessary for the know-how of the target audience	0.92%
3.5 It is suitable for use by the target audience of ET	0.75%
IVC Global	0.85%

*CVI - Content Validity Index; ET - Educational Technology; N - 19 judges.*

Chart 1 presents the expert judges’ suggestions regarding the content, presentation and structure of the educational booklet:

The overall analysis of the booklet by the target audience obtained an assessment of the level of agreement above 85%, which was considered an excellent evaluation by the research participants. Only 25% of the interviewees described their personal opinions in the booklet evaluation instrument, according to item 6 - personal opinions. Of the participants, 4 patients described that “a booklet should be given to each patient”, “this booklet should be given to all hospitals”. 3 patients described that the booklet is too long and only 2 patients reported “not having any hope of improving health care in Brazil”. Table 2 presents the evaluation items by the target audience.

**Table 2:** Evaluation of the booklet by the target audience Contents, Manaus, Amazonas, Brazil, 2022

Factor to be examined	Yes n(%)	No n(%)	Partially n(%)
1. Literary Presentation			
1.1 The language of the booklet is explanatory;	36(100%)	^*^	^*^
1.2 The material promotes and encourages adherence to patient safety measures;	36(100%)	^*^	^*^
1.3 The vocabulary is mostly composed of simple and common language;	36(100%)	^*^	^*^
1.4 The signaling of the title and subtitles aids in learning;	36(100%)	^*^	^*^
1.5 The language is appropriate for the target audience;	36(100%)	^*^	^*^
1.6. The text presents a logical sequence of patient safety precautions;	36(100%)	^*^	^*^
1.7 The material is pleasant to read;	36(100%)	^*^	^*^
1.8 The material is of adequate size, is not extensive or tiring;	36(100%)	^*^	^*^
1.9 The booklet, in general, is simple and attractive.	36(100%)	^*^	^*^
2. Illustrations			
2.1 The illustrations are simple, appropriate and have easy-to-understand lines;	36(100%)	^*^	^*^
2.2. They are familiar to readers;	6(16.66%)		30(8.33%)
2.3 They are related to the text (they configure the desired purpose);	36(100%)	^*^	^*^
2.4 Estão integradas ao texto;	36(100%)	^*^	^*^
2.5 As figuras são autoexplicativas.	2(5.56%)	^*^	34(94.44%)
3. Sufficient expressive and comprehensive material.			
3.1 Provides maximum understanding for preventing adverse events in pa-tient safety;	36(100%)	^*^	^*^
3.2 The instructions for care safety are clear and comprehensive;	36(100%)	^*^	^*^
3.3 The text does not allow for ambiguous meaning;	36(100%)	^*^	^*^
3.4 The content is written in a style with the target audience as the center, that is, the patient is the most important;	36(100%)	^*^	^*^
4. Legibility and print characteristics.			
4.1 Is the cover attractive?	36(100%)	^*^	^*^
4.2 Does the cover convey the subject matter?	36(100%)	^*^	^*^
4.3 Is the font size adequate;	1(2.78%)	^*^	35(97.22%)
4.5 Is the spacing of letters adequate;	1(2.78%)	^*^	35(97.22%)
4.6 Is the font style adequate;	36(100%)	^*^	^*^
4.7 Is the length between lines adequate;	36(100%)	^*^	^*^
4.8 Does the use of boldface and bullet points draw attention to specific points or key content;	36(100%)	^*^	^*^
4.9 Is there adequate use of white space to reduce the appearance of clut-tered text;	36(100%)	^*^	^*^
4.10 Is there good contrast between the printing and the paper;	36(100%)	^*^	^*^
4.11 Does the paper used facilitate viewing;	36(100%)	^*^	^*^
4.12 Do subtitles or entries facilitate understanding and memorization;	36(100%)	^*^	^*^
4.13 Is the spacing between paragraphs adequate;	36(100%)	^*^	^*^
4.14 Is the format of the material adequate.	36(100%)	^*^	^*^
5. Quality of Information.			
5.1 The booklet is part of the local culture;	9(25%)	^*^	27(75%)
5.2 The booklet is part of the current culture;	1(2.78%)	^*^	35(97.22%)
5.3 The material enables the target audience to perform the desired actions;	36(100%)	^*^	^*^
5.4 The material helps prevent potential problems;	36(100%)	^*^	^*^
5.5 The material allows for maximum benefit;	36(100%)	^*^	^*^
5.6 The use of the booklet is relevant;	36(100%)	^*^	^*^
5.7 The booklet proposes that the learner acquire knowledge;	36(100%)	^*^	^*^

**Chart 1:** Suggestions provided by judges in the process of validating the educational booklet

Suggestions regarding content	Situation
Replacing repeated phrases;	accepted
Correction of Portuguese errors;	accepted
Addition of new suggested texts (items Falls, Safe administration of medication, Safe surgery and Hand hygiene);	accepted
Changes to texts for better understanding in injury prevention (ADD the three pieces of information): Keep your skin clean and moisturized; Always change diapers and use moisturizers; Protect the fragile parts of your body; Take care of your diet and hydration.	accepted
**Suggestions regarding presentation and structure**	**Situation**
Page number ingchanges.	accepted

### Final version of educational technology

**Figure 1:**
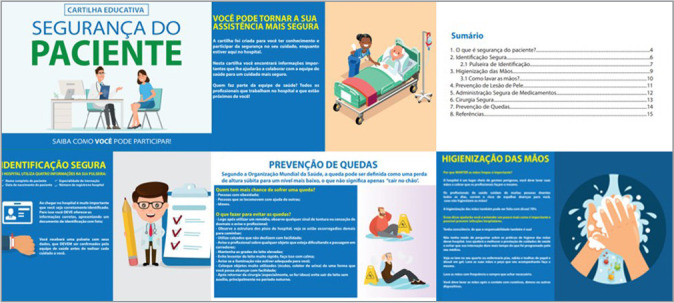
Cover, presentation, summary and parts of the educational booklet produced and validated in this study, Manaus, Amazonas, Brazil, 2022

The booklet was formatted with a total of 15 pages on A4 paper (210 x 297 mm), in landscape orientation. Two pages consist of pre-textual elements, twelve with textual elements and one post-textual page. The material is organized by Patient Safety Protocol: Patient Identification; Hand Hygiene; Skin Injury Prevention; Safe Medication Administration; Safe Surgery; Fall Prevention. In Figure 1, there are some pages of the educational booklet. To identify the booklet, we used the International Standard Book Number (ISBN), represented by the Code 978-65-00-64926-0.

## DISCUSSION

Knowledge and understanding of the experiences of patients and family members when adverse events occur provide important information for strengthening the safety culture within the organizational context. Recognizing that patients are the holders of knowledge is essential for the effectiveness and safety of treatment^(18)^. Sharing information and open communication are one of the changes in the patient safety culture, not based on patient ignorance or stigma^(19)^.

Patient involvement in care safety has been understood as a means of reducing risks associated with health care, depending, of course, on the type of collaboration that patients can establish with professionals^(20)^. Patients and family members who participate in care become more active and engaged in identifying unsafe situations before incidents occur. In addition, participation in care contributes to the safe use of medication, resulting from knowledge of the medications used and the possible effects or adverse events, as well as helping to control infection, encourage hand washing and effectively communicate complications and adverse events in favor of a non-punitive culture of organizational learning^(21)^.

The presentation of the booklet is effective in promoting improvements in the health area^(22)^. The contents covered in the booklet contribute to the dissemination of important information recommended by the World Health Organization in the six international patient safety goals. Thus, through the booklet with the layout of the content in text boxes, topics and illustrations, the material becomes easier to understand during reading, as demonstrated during the interviews.

The educational material was considered understandable and attractive, characterized as alternatives to raise patient awareness so that they can participate in their self-care^(23)^. In the present study, it was possible to prove the feasibility of using the booklet, as patients and companions expressed interest in reading the material and learning about the six Patient Safety goals. The patient’s perception of safety can influence their engagement and that of their family members regarding safe practices^(24)^. Contributing factors related to communication, identification and hand hygiene emerged in the patients’ reports, which were related to the six safety goals.

The educational booklet was not only created to replace the verbal instructions given by the multidisciplinary team during care, but also to reinforce the instructions and resolve doubts, since the patient can consult the material whenever he/she is interested^(25)^. It is therefore suggested that the team use the educational material during educational strategies, making the technology facilitate the construction of knowledge between health professionals and patients and family members, in addition to helping to clarify doubts^(26)^.

Disseminating printed educational materials is effective in contributing to improvements in the health area. It is therefore pertinent to develop methodological studies that contemplate the construction and validation of educational health materials for provision in health services^(27)^. Paradoxically, in the current Brazilian context of lack of supplies and precariousness of the structure of hospital services, giving patients a voice is both urgent and necessary to the founding principles of the Unified Health System.

Therefore, the educational technology presented here was considered validated and capable of bringing patients closer to professionals and consolidating health care in a way that increases the patient’s ability to contribute to their treatment and prevent adverse events from occurring.

### Study limitations

One of the limitations was the COVID-19 (Coronavirus) pandemic, which delayed the application of the booklet to study patients, with a greater risk of contamination of researchers and patients, while maintaining current sanitary measures.

### Contributions to the field of nursing

Nursing plays a fundamental role in care since it is constantly close to the patient. Care needs to be systematized and conditioned between the health team and the user to ensure the quality of care^(28)^.

## FINAL CONSIDERATIONS

It is believed that this educational technology can serve as essential support for the healthcare team. Professionals have the possibility of working on positioning the patient as an active participant in their own safety, since this new condition facilitates care and a closer relationship between user and professional.

The use of this booklet can more assertively achieve an organizational safety culture, since it encompasses the active participation of the patient. However, there is still resistance to the insertion of tools as they were presented, without their full potential being used. To this end, persistence in using the material can contribute to safer care, a well-informed patient and family member, and an institution that stands out in the aspect of Patient Safety.
