# Supplementary material for: Educational technology to empower patients as participants in their care
Source: Rev Bras Enferm. 2024 Dec 16;77(6):e20230359. doi: 10.1590/0034-7167-2023-0359 (PMC11654525; doi:10.1590/0034-7167-2023-0359)
Supplement: Translation

# Translation: Tecnologia educacional para empoderamento do paciente como partícipe do seu cuidado

**DOI:** 10.1590/0034-7167-2023-0359pt

**Authors:** Rodrigo da Silva Ramos, Mateus Ferreira de Aguiar, Eurinete Catarina Guimarães da Silva, Cleberson Morais Caetano, Rizioléia Marina Pinheiro Pina, Joice Claret Neves, Hadelândia Milon de Oliveira

**Affiliations:** I Secretaria Municipal de Saúde de Manaus. Manaus. Amazonas. Brasil; II Universidade Estadual do Amazonas. Manaus. Amazonas. Brasil; III Universidade Federal do Amazonas. Manaus. Amazonas. Brasil; IV Empresa Brasileira de Serviço Hospitalares. Florianópolis. Santa Catarina. Brasil

**Keywords:** Segurança do Paciente, Educação em Saúde, Tecnologia Educacional, Pacientes, Enfermagem.

## Abstract

**Objetivos:**

construir e validar uma tecnologia educativa para o empoderamento do paciente como partícipe do seu cuidado.

**Métodos:**

estudo metodológico. para desenvolvimento de uma tecnologia educativa a partir da elaboração. validação e avaliação que foram realizadas em cinco etapas. em um Hospital de ensino no Amazonas. O estudo foi realizado no período de 2019 a 2022. participaram 19 juízes especialistas na temática de segurança do paciente e72 pacientes internados nas clínicas médicas e cirúrgicas do hospital. cenário do estudo.

**Resultados:**

a concordância entre os juízes obteve índice global de 0.85. sendo considerado validado. A análise global da cartilha obteve uma avaliação do nível de concordância acima de 85%.

**Considerações Finais:**

a tecnologia educativa aqui apresentada foi validada e apta para promover a aproximação paciente/profissional e consolidar a assistência em saúde de uma maneira que aumente a capacidade do paciente para contribuir no seu tratamento e evitar a ocorrência de eventos adversos.

## INTRODUÇÃO

A segurança do paciente teve seu conceito ampliado. definida como uma estrutura de atividades organizadas que cria culturas. processos. procedimentos comportamentos. tecnologias e ambientes na área da saúde com riscos mais baixos de forma consistente e sustentável. para mitigar a ocorrência de danos evitáveis e reduzir seus impactos quando ocorrem^(1)^.

Estima-se que. em países de baixa renda. um em cada 4 pacientes está exposto a um incidente com dano nos serviços de saúde^(2)^. Em média 60% das mortes acontecemem razão de práticas inseguras e de má qualidade da assistência prestada^(3)^. Os eventos adversos são decorrentes de erros sistêmicos ou organizacionais que favorecem a ocorrência de falha na assistência prestada^(4)^.

Contudo. se a garantia da qualidade em saúde for somente por meio do resultado. a alta gestão centralizará esforços em processos. ações e produtos. o que poderá depreciar relacionamentos e preferências individuais do paciente. Portanto. repensar o cuidado em saúde como coprodução pode trazer benefícios para melhoria da qualidade nos serviços de saúde^(5)^.

O plano de ação global para segurança do paciente 2021-2030. em busca da eliminação dos danos evitáveis nos cuidados de saúde. dispõe de sete princípios norteadores para balizar ações. tendo em dois desses princípios o paciente como foco em prol do cuidado em saúde livre de danos. a saber: engajar paciente e familiar enquanto parceiros no cuidado seguro e utilização da experiência do paciente para aprimorar a segurança^(6)^.

Nesse contexto. conhecer a visão do paciente e seus familiares vem sendo prioridade. inclusive para ajudar a construir processos de cuidados centrados no paciente e melhoria do desempenho das equipes clínicas^(7)^. Portanto. a equipe de saúde com precisão deve orientar os pacientes. utilizando materiais educativos que facilitem a sua compreensão.

Pacientes e acompanhantes. devidamente orientados e empoderados pelo autocuidado. são capazes de relatar. sem prejuízo. incidentes e os fatores contribuintes. fornecendo informações novas e valiosas sobre o tipo e a frequência dessas ocorrências. o que possibilita a melhoria da qualidade da assistência. com embasamento para tomadas de decisões compartilhadas e assertivas^(8)^.

O uso de tecnologias educativas impressas. como álbum seriado e cartilhas. é uma alternativa viável para informação e sensibilização dos pacientes. para promoção a saúde. além de permitir uma leitura posterior. que reforça as orientações verbais. servindo como guia em casos de dúvidas^(9)^.

A elaboração. validação e implementação de materiais educativos do tipo cartilha tem sido reincidente em estudos nacionais e internacionais. sendo importante que as ferramentas educativas sobre segurança do paciente sejam desenvolvidas e validadas para utilização na prática clínica com intuito de contribuir na disseminação da cultura de segurança do paciente. nos serviços de saúde^(10)^. A cartilha é um mecanismo considerado de maior viabilidade financeira na disseminação de informação^(11)^.

Sendo assim. é relevante discutir sobre as metodologias de ensino que sejam capazes de auxiliar pacientes e familiares no seu autocuidado. facilitando a compreensão sobre os pilares da segurança do paciente.

A cartilha educativa entra como potencial aliado para clarificar idéias e como fonte de informação para pacientes. Para a segurança do paciente. essa tecnologia tem capacidade de norteá-lo acerca da internação hospitalar além de guiá-lo no seu engajamento para uma assistência segura^(12)^.

Diante do exposto. destaca-se a importância deste estudo para construção. validação e avaliação de uma Tecnologia Educacional. por contemplar três eixos da Segurança do Paciente. a saber: estimula prática assistencial segura. envolvimento do cidadão na segurança e promover pesquisa sobre segurança do paciente^(13)^.

## OBJETIVOS

Construir e validar uma tecnologia educativa para o empoderamento do paciente como partícipe do seu cuidado seguro

## MÉTODOS

### Aspectos éticos

A pesquisa foi aprovada pelo Comitê de Ética em Pesquisa da Universidade Federal do Amazonas (UFAM). por meio da Plataforma Brasil. em concordância com a Resolução 466/2012 (CNS. 2012). Tanto nas fases de validação. realizadas pelos juízes especialistas. quanto nas de avaliação. feitas pelos pacientes. todos foram informados sobre os objetivos do estudo e aceitaram participar através do Termo de Consentimento Livre e Esclarecido (TCLE). assinado em duas vias. ficando uma com cada participante e outra com a equipe da pesquisa.

### Referencial teórico-metodológico

A construção da cartilha educativa (CE) foi pautada pelos conceitos do modelo Andragógico que. por ser flexível. pode ou não ser adotado por completo. pois a força da Andragogia “reside em um conjunto de seis princípios fundamentais sobre a Aprendizagem de Adultos que se aplicam a todas as situações de aprendizagem”^(14)^.

Tem-se como premissas: 1. A necessidade de conhecer: o adulto precisa saber por que deverá aprender algo antes de começar a aprender de fato; 2. O autoconceito do aprendiz: o adulto se vê como responsável por sua própria vida e por suas decisões. e quer ser visto e tratado pelos outros como capaz de se autodirigir; 3. O papel da experiência: o adulto acumula um conjunto de experiências que se torna uma fonte inesgotável de aprendizagem; 4. Prontidão para aprender: o adulto se engaja em aprender aquilo que poderá ajudá-lo a solucionar problemas da vida real e a desempenhar seus papéis sociais; 5. Orientação para aprendizagem: a perspectiva temporal do adulto está focada na aplicação imediata do conhecimento; 6. Motivação: fatores extrínsecos e fatores intrínsecos^(14)^.

No contexto da segurança do paciente. em que o adulto se encontra em situação de hospitalização.entende-se que a Andragogia é capaz de designar princípios fundamentais para contribuir com o processo de ensino-aprendizagem de forma crítica e reflexiva. para o fortalecimento do aprendizado e empoderamento do paciente como partícipe do seu cuidado.

### Tipo de estudo

Trata-se de um estudo metodológico de construção de uma CE de validação. com juízes e avaliação com o público alvo. norteado pela ferramenta Consolidated Criteria for Reporting Qualitative Research (COREQ). disponibilizada pela Rede EQUATOR. que orienta a descrição de estudos qualitativos^(15)^.

### Procedimentos metodológicos

O estudo foi realizado em cinco etapas: identificação do conhecimento de usuários de um hospital-escola acerca dos cuidados que contribuem para a segurança do paciente; revisão de literatura; construção da CE; validação de conteúdo e aparência da CE pelos juízes; avaliação do conteúdo e aparência da CE pelo público-alvo^(16)^.

Na etapa 1.realizou-se o estudo descritivo-qualitativo para identificar o conhecimento de usuários de um hospital-escola acerca dos cuidados que contribuem para a segurança do paciente^(17)^. relevante para o entendimento do conhecimento do sujeito da pesquisa sobre a segurança do paciente no ambiente hospitalar para direcionar a construção da CE.

Na etapa2. foi realizada uma pesquisa bibliográfica. registrada na plataforma PROSPERO e desenvolvida pelo método do tipo Scoping Review. Construiu-se a questão norteadora utilizando como estratégia a combinação mnemônica PCC: P *Population* - pacientes adultos; C *Concept* - tecnologias educacionais em saúde; C *Context* - segurança do paciente. Dessa forma. definiu-se a seguinte pergunta de pesquisa: quais e como estão sendo aplicadas as tecnologias educacionais em saúde ao paciente no contexto da sua própria segurança?

Foi realizada a pesquisa nas bases de dados via periódicos da Coordenação de Aperfeiçoamento de Pessoal de Nível Superior (Capes): Biblioteca Virtual de Saúde (BVS). Literatura Latino-Americana e do Caribe em Ciências da Saúde (LILACS). *Medical Literature Analysis and Retrieval System Online* (MEDLINE) e *Scientific Electronic Library Online* (SCIELO). Public/Publisher MEDLINE (PubMed). SciVerse SCOPUS e Base de Dados em Enfermagem (BDENF).Foram incluídos na pesquisa artigos nos idiomas em português. espanhol e inglês. publicados no período entre 2010 e 2020. que estavam disponíveis para leitura na íntegra.

Na etapa 3ocorreu a elaboração da cartilha. realizada no período de maio a agosto de 2020. sendo consideradas as falas dos pacientes entrevistados e a realização de um levantamento bibliográfico acerca das recomendações sobre segurança do paciente de órgãos internacionais (Organização Mundial da Saúde e *Pan American Health Organization*). Manuais do Ministério da Saúde e os resultados da revisão de escopo.

Foram realizadas a elaboração do conteúdo. a ilustração e a diagramação da tecnologia educacional. juntamente com os pesquisadores envolvidos no estudo e um profissional de programação gráfica/design. com observância dos critérios relacionados ao conteúdo. estrutura. organização. linguagem. layout. design. sensibilidade cultural e adequação ao paciente em situação de internação hospitalar. com apresentação prévia aos enfermeiros membros do Núcleo de Segurança do paciente do Hospital Universitário. local onde. posteriormente. foi aplicada a cartilha.

Na etapa 4 ocorreu a validação da Tecnologia educativa pelo Índice de Validade de Conteúdo por juízes experts. no período de fevereiro a agosto de 2020. No contato inicial. os juízes experts receberam o convite para participar da pesquisa contendo as instruções e o Termo de Consentimento Livre e Esclarecido (TCLE). Após o aceite. os juízes experts receberam a Escala de *Likert* e a cartilha educativa.

Na etapa 5. realizou-se a avaliação do conteúdo e da aparência da cartilha educativa pelo público-alvo. aplicada individualmente a cada paciente. Após assinatura do TCLE. cada paciente recebeu um exemplar da cartilha educativa segurança do paciente. Após a leitura. o instrumento de avaliação foi entregue aos pacientes. sendo respondido entre 15 e 20 minutos. A coleta de dados ocorreu no período de maio a junho de 2022.

### Ambiente de estudo

O estudo foi realizado em um hospital-escola vinculado a UniversidadeFederal. referência no atendimento de média e alta complexidade em toda a Amazônia Ocidental. atuando também na formação de profissionais de saúde de graduação e pós-graduação e desenvolvimento de pesquisas científicas e extensão universitária.

### Fonte de dados

Para a validação de conteúdo. foram convidados 20 juízes especialistas. destes. 19 aceitaram participar da pesquisa. Utilizou-se como critério de elegibilidade: a) ter experiência de no mínimo 3 anos em segurança do paciente e/ou atendimento ao paciente clínico ou cirúrgico internado; b) possuir no mínimo uma especialização *lato sensu* em saúde. O levantamento dos juízes experts elegíveis foi feito pela Plataforma *Lattes* do portal do Conselho Nacional do Desenvolvimento Científico e Tecnológico (CNPq). utilizando os seguintes filtros: segurança do paciente e com especialização no assunto. Apenas um foi excluído porque respondeu o questionário acima de 30 dias.

Para a avaliação do conteúdo e aparência pelo público-alvo. participaram 36 pacientes. Utilizou-se como critérios de seleção: pacientes internados pela primeira vez nas unidades clínica médica e cirúrgica. independentemente da idade. sexo e diagnóstico médico. com permanência mínima de 24 horas de internação. Foram excluídos os pacientes que se encontravam com rebaixamento do nível de consciência. clinicamente instável e os pacientes com programação de transferência interna ou externa para outras unidades clínicas. no período da coleta de dados. O levantamento do público-alvo foi diretamente nas unidades de internação. por levantamento prévio dos dados clínicos do paciente nos prontuários. em seguida houve abordagem direta ao paciente elegível no estudo. Justifica-se a inclusão dos pacientes internados. uma vez que esses pacientes vivenciam experiência que requerem cuidados seguros com menor risco de falhas na assistência prestada. durante o tempo de internação hospitalar. e seu envolvimento nos cuidados é imprescindível.

### Coleta e organização dos dados

Na etapa 4 de validação da Tecnologia educativa pelo Índice de Validade de Conteúdo por juízes experts o instrumento utilizado possui três partes: identificação. instruções e blocos de questões com a seguinte estrutura: a) objetivos; b) estrutura e apresentação; c) relevância; d) comentários gerais e sugestões. A Escala *Likert* foi constituída dos seguintes itens: Totalmente Adequado (TA); Adequado (A); Parcialmente Adequado (PA); Inadequado (I).

O prazo de devolutiva pelos juízes foi de até 15 dias. Aos que não devolveram a escala nos períodos propostos. tiveram mais 7 dias para enviá-la e. por fim. aqueles que não enviaram foram excluídos da pesquisa.

Na etapa 5. foi realizada a avaliação do conteúdo e aparência da cartilha educativa pelo público-alvo. utilizando um Instrumento contendo 6 itens: 1. Apresentação Literária; 2. Ilustrações; 3. Material suficiente expressivo e compreensivo; 4. Legibilidade e características da impressão; 5. Qualidade da Informação; 6. Opiniões pessoais (esta última com aspecto descritivo). Com respostas de cada item. sendo: “Sim”. “Não” ou “Em Parte”.

### Análise dos dados

Para a validação de conteúdo. calculou-se o escore do Índice de Validade de Conteúdo (IVC). o que permite analisar cada item separadamente e como um todo. A análise estatística utilizou o percentual de resposta válida para cada item. a validação se deu por bloco. ou seja. a média de resposta para cada item.

Na validação pelos juízes. foram utilizadas as somas das respostas referentes aos itens “Totalmente Adequado” e “Adequado”. Após esse cálculo. o IVC mínimo esperado era maior ou igual 0.70 ou 70% de aprovação. A concordância entre os juízes obteve IVC global de 0.85 na primeira avaliação. sendo considerado validado.

As sugestões dos juízes especialistas com relação à primeira versão da cartilha foram atendidas no que se refere a: troca de frases repetidas. correção de erros de português. mudanças na numeração de página. mudanças de textos para melhor compreensão e adição de textos novos sugeridos (nos itens Quedas. Administração Segura de Medicamentos. Cirurgia Segura e Higienização das mãos).

A avaliação pelo público-alvo foi realizada com as respostas de cada item. sendo “Sim”. “Não” ou “Em Parte”. Considerados válidos os itens com resposta “Sim” ou “Em Parte”. Os itens com concordância mínima de 75% foram considerados validados. Os itens com índice de concordância menor foram considerados passíveis de alteração.

## RESULTADOS

Na primeira etapa do estudo descritivo-qualitativo. para identificar o conhecimento de usuários de um hospital-escola acerca dos cuidados que contribuem para a segurança do paciente. foram entrevistados 72 pacientes. sendo 52 (72.2%) do sexo feminino e 20 (27.8%) do sexo masculino com maior taxa de participação entre 41-46 anos. Quanto à clínica de internação. 6 (8.3%) estavam internados na Clínica Médica e 66 (91.7%) na Clínica Cirúrgica do Hospital Universitário pesquisado.Pela análise de Bardin emergiram 3 categorias: “Cuidado e Segurança do Paciente”; “Diálogo com a equipe”; “Confirmação dos procedimentos e confiança”^(17)^.

A Revisão bibliográfica apontou diferentes tipos e métodos de Tecnologias Educacionais aplicadas aos pacientes e familiares para o envolvimento no seu cuidado seguro. podendo se apresentar de diversas formas - das mais simples e tradicionais até as digitais e com maiores recursos tecnológicos de disseminação de conhecimento de forma remota. eficientes no contexto da pandemia do novo Coronavírus.

Na validação da tecnologia. participaram 19 juízes especialistas. a maioria do sexo feminino (76.7%). Em relação à formação profissional. 89.7% eram enfermeiros e 10.3% farmacêuticos. Quanto à titulação. 51.7% eram doutores. 10.3% mestres e 38% especialistas.

Participaram das entrevistas da etapa de avaliação da cartilha pelo público-alvo 36 pacientes internados nas clínicas médicas (32%) e cirúrgicas (68%). todos com primeira internação. independente de sexo. diagnóstico e com permanência mínima de 24 horas de internação.

A concordância entre os juízes obteve IVC global de 0.85 na primeira avaliação. sendo considerado validado. Foram utilizadas as somas das respostas referentes aos itens Totalmente Adequado e Adequado. A Tabela 1 apresenta os índices individual e global de cada item avaliado.

**Tabela 1:** Avaliação da concordância da adequação da cartilha educativa quanto ao Índice de Validade de Conteúdo, Manaus, Amazonas, Brasil, 2022

Domínio / Itens de avaliação	IVC N19(%)
1. Objetivo (IVC)	0,86%
1.1 As informações/conteúdos são importantes para a qualidade do trabalho/qualidade de vida do público-alvo da TE	0,96%
1.2 Convida e/ou instiga a mudanças de comportamento e atitude	0,89%
1.3 Pode circular no meio científico da área	0,82%
1.4 Atende aos objetivos de instituições em que trabalha/atende o público-alvo da TE	0,75%
2. Estrutura e Apresentação (IVC)	0,83%
2.1 A TE é apropriada para o público	0,86%
2.2 As mensagens estão apresentadas de maneira clara e objetiva	0,68%
2.3 As informações apresentadas estão cientificamente corretas	0,93%
2.4 O material está apropriado ao nível sociocultural do público-alvo da TE	0,78%
2.5 Há uma sequência lógica do conteúdo proposto	0,93%
2.6 As informações estão bem estruturadas em concordância e ortografia	0,79%
2.7 O estilo da redação corresponde ao nível de conhecimento do público-alvo	0,78%
2.8 As informações da capa. contracapa. sumário. agradecimentos e/ou apresentação estão coerentes	0,80%
2.9 O tamanho do título e dos tópicos está adequado	0,72%
2.10 As ilustrações estão expressivas e suficientes	0,96%
2.11 O material (papel/impressão) está apropriado	NA
2.12 O número de páginas está adequado	0,89%
3. Relevância (IVC)	0,88%
3.1 Os temas retratam aspectos-chave que devem ser reforçados	1,00%
3.2 A TE permite a transferência e generalização do aprendizado a diferentes contextos	0,78%
3.3 A TE propõe a construção do conhecimento	0,96%
3.4 A TE aborda os assuntos necessários para o saber-fazer do público-alvo	0,92%
3.5 Está adequada para ser usada pelo público-alvo da TE	0,75%
IVC Global	0,85%

*IVC - Índice de Validade de Conteúdo; TE - Tecnologia Educacional; N - 19 juízes.*

No Quadro 1. apresentam-se as sugestões dos juízes especialistas referentes ao conteúdo e à apresentação e estrutura da cartilha educativa.

**Tabela 2:** Avaliação da cartilha pelo público-alvo Conteúdos. Manaus. Amazonas. Brasil. 2022

Fator a ser examinado	Sim n(%)	Não n(%)	Em parte n(%)
1. Apresentação Literária			
1.1 A linguagem da cartilha é explicativa;	36(100%)	^*^	^*^
1.2 O material promove e encoraja a adesão as medidas de segurança do paciente;	36(100%)	^*^	^*^
1.3 O vocabulário em sua maioria é composto por linguagem simples e comuns;	36(100%)	^*^	^*^
1.4 A sinalização do título e subtítulos auxilia na aprendizagem;	36(100%)	^*^	^*^
1.5 A linguagem está adequada ao público-alvo;	36(100%)	^*^	^*^
1.6. O texto apresenta sequência lógica dos cuidados para segurança do paciente;	36(100%)	^*^	^*^
1.7 O material é de leitura agradável;	36(100%)	^*^	^*^
1.8 O material tem tamanho adequado. não é extenso e nem cansativo;	36(100%)	^*^	^*^
1.9 A cartilha. de um modo geral. é simples e atrativa.	36(100%)	^*^	^*^
2. Ilustrações			
2.1 As ilustrações são simples. apropriadas e de tracejado de fácil compreensão;	36(100%)	^*^	^*^
2.2. São familiares para os leitores;	6(16.66%)		30(8.33%)
2.3 Estão relacionadas com o texto (configuram o propósito desejado);	36(100%)	^*^	^*^
2.4 Estão integradas ao texto;	36(100%)	^*^	^*^
2.5 As figuras são autoexplicativas.	2(5.56%)	^*^	34(94.44%)
3. Material suficiente expressivo e compreensivo.			
3.1 Propicia o máximo de compreensão para prevenção de eventos adversos na segurança do paciente;	36(100%)	^*^	^*^
3.2 As instruções para a segurança do cuidado são claras e compreensivas;	36(100%)	^*^	^*^
3.3 O texto não possibilita sentido dúbio;	36(100%)	^*^	^*^
3.4 O conteúdo é escrito em estilo ao público-alvo como centro. ou seja. o paciente é o mais importante;	36(100%)	^*^	^*^
4. Legibilidade e características da impressão.			
4.1 A capa é atraente?	36(100%)	^*^	^*^
4.2 A capa mostra o assunto a que se refere?	36(100%)	^*^	^*^
4.3 O tamanho da letra é adequado;	1(2.78%)	^*^	35(97.22%)
4.5 O espaçamento das letras é adequado;	1(2.78%)	^*^	35(97.22%)
4.6 O estilo da letra é adequado;	36(100%)	^*^	^*^
4.7 O comprimento entre as linhas é adequado;	36(100%)	^*^	^*^
4.8 A utilização de negrito e marcadores de texto chamam atenção dos pontos específicos ou conteúdos chaves;	36(100%)	^*^	^*^
4.9 Existe uso adequado de espaço em branco para reduzir a aparência do texto abarrotado;	36(100%)	^*^	^*^
4.10 Existe bom contraste entre a impressão do papel;	36(100%)	^*^	^*^
4.11 O papel utilizado facilita a visualização;	36(100%)	^*^	^*^
4.12 Os subtítulos ou as entradas facilitam a compreensão e memorização;	36(100%)	^*^	^*^
4.13 O espaçamento entre os parágrafos é adequado;	36(100%)	^*^	^*^
4.14 O formato do material é adequado.	36(100%)	^*^	^*^
5. Qualidade da Informação.			
5.1 A cartilha está inserida na cultura local;	9(25%)	^*^	27(75%)
5.2 A cartilha está inserida na cultura atual;	1(2.78%)	^*^	35(97.22%)
5.3 O material habilita o público-alvo a realizar as ações desejadas;	36(100%)	^*^	^*^
5.4 O material ajuda a prevenir possíveis problemas;	36(100%)	^*^	^*^
5.5 O material permite obter o máximo benefício possível;	36(100%)	^*^	^*^
5.6 O uso da cartilha se faz relevante;	36(100%)	^*^	^*^
5.7 A cartilha propõe ao aprendiz adquirir conhecimento;	36(100%)	^*^	^*^

**Quadro 1:** Sugestões fornecidas pelos juízes no processo de validação da cartilha educativa

Sugestões referentes ao conteúdo	Situação
Troca de frases repetidas;	acatada
Correção de erros de português;	acatada
Adição de textos novos sugeridos (itens Quedas. Administração segura de medicamentos. Cirurgia Segura e Higienização das mãos);	acatada
Mudanças de textos para melhor compreensão na prevenção de lesão (ACRESCENTAR as três informações): Manter a pele limpa e hidratada; Sempre trocar as fraldas e usar hidratantes; Proteja as partes frágeis do seu corpo; Cuide da alimentação e de sua hidratação.	acatada
**Sugestões referentes à apresentação e estrutura**	**Situação**
Mudanças na numeração de página.	acatada

A análise global da cartilha pelo público-alvo obteve uma avaliação do nível de concordância acima de 85%. sendo considerada como uma ótima avaliação pelos participantes da pesquisa. Apenas 25% dos entrevistados descreveram suas opiniões pessoais no instrumento de avaliação da cartilha. conforme o item 6 - opiniões pessoais. Dos participantes. 4 pacientes descreveram que “deveria ser entregue uma cartilha a cada paciente”. “esta cartilha deveria ser entregue em todos os hospitais”. 3 pacientes descreveram que a cartilha está muito extensa e apenas 2 pacientes informaram “não ter esperança de melhoria na saúde do Brasil”. A Tabela 2 apresenta os itens de avaliação pelo público-alvo.

### Versão final da tecnologia educativa

A cartilha foi formatada com um total de 15 páginas em folha A4 (210 x 297mm). na orientação paisagem. Duas páginas consistem em elementos pré-textuais. doze com elementos textuais e uma página pós-textual. O material está organizado por Protocolo de Segurança do Paciente: Identificação do Paciente; Higiene das Mãos; Prevenção de Lesão de Pele; Administração Segura de Medicamentos; Cirurgia Segura; Prevenção de Quedas. Na Figura 1. apresentam-se algumas páginas da cartilha educativa. Para identificação da cartilha. utilizamos o International Standard Book Number (ISBN). representado pelo Código 978-65-00-64926-0.

**Figura 1:** Capa. apresentação. sumário e partes da cartilha educativa produzida e validada nesse estudo. Manaus. Amazonas. Brasil. 2022

## DISCUSSÃO

O conhecimento e compreensão das experiências de pacientes e familiares quando ocorrem eventos adversos fornecem informações importantes para o fortalecimento da cultura da segurança no âmbito organizacional. O reconhecimento de que os pacientes são detentores de conhecimento é essencial para a efetividade e segurança do tratamento^(18)^. O compartilhamento de informações e a comunicação aberta são uma das mudanças na cultura de segurança do paciente. não se baseando na ignorância ou estigma do paciente^(19)^.

O envolvimento do paciente na segurança do cuidado vem sendo compreendido como meio de reduzir riscos associados aos cuidados de saúde. dependente. claro. do tipo de colaboração que pacientes possam estabelecer com os profissionais^(20)^. Pacientes e familiares participativos no cuidado tornam-se mais ativos e engajados na identificação de situações inseguras antes que incidentes ocorram. Além disso. a participação no cuidado contribui para o uso seguro da medicação. advinda do conhecimento daqueles medicamentos utilizados e dos possíveis efeitos ou eventos adversos. assim como colabora no controle de infecção. no estímulo a lavagem das mãos e na comunicação efetiva de complicações e eventos adversos em prol de uma cultura não punitiva do aprendizado organizacional^(21)^.

A apresentação da cartilha é eficaz para promover melhorias na área da saúde^(22)^. Os conteúdos abordados na cartilha contribuem para divulgação de informações importantes preconizadas pela Organização Mundial da Saúde nas seis metas internacionais da segurança do paciente. Assim. através da cartilha com a diagramação do conteúdo em caixas de texto. tópicos e ilustrações. o material fica mais fácil para ser compreendido durante a leitura. como foi demonstrado durante as entrevistas.

O material educativo foi considerado compreensível e atrativo. caracterizado como alternativas de sensibilização do paciente de forma que este possa ser participativo no seu autocuidado^(23)^. No presente estudo. foi possível comprovar a viabilidade para utilização da cartilha. pois os pacientes e acompanhantes manifestaram interesse em ler o material e aprender sobre as 6 metas de Segurança do Paciente. A percepção de segurança pelo paciente pode influenciar o seu engajamento e de seus familiares quanto as práticas seguras^(24)^. Fatores contribuintes relacionados à comunicação. identificação e higienização das mãos emergiram nos relatos dos pacientes. sendo estes relacionados às seis metas de segurança.

A cartilha educativa não foi construída apenas com intuito de substituir as orientações verbais da equipe multiprofissional durante a assistência. mas também para reforçar as orientações e solucionar dúvidas. pois o paciente poderá consultar o material sempre que tiver interesse^(25)^. Sugere-se. então. que a equipe faça uso do material didático durante as estratégias educativas. fazendo com que a tecnologia facilite a construção de conhecimento entre os profissionais da saúde com o paciente e familiares. além de auxiliar no esclarecimento de dúvidas^(26)^.

A divulgação de materiais educativos impressos é eficaz para contribuir com melhorias na área da saúde. Torna-se. então. pertinente o desenvolvimento de estudos metodológicos que contemplem a construção e validação de materiais educativos em saúde para disponibilização em serviços de saúde^(27)^. Paradoxalmente. no contexto atual brasileiro de carência de insumos e precarização da estrutura dos serviços hospitalares. dar voz aos pacientes é tanto urgente como necessário aos princípios fundantes do Sistema Único de Saúde.

Dessa forma. a tecnologia educativa aqui apresentada foi considerada validada e apta para aproximar o paciente do profissional e consolidar a assistência em saúde de uma maneira que aumente a capacidade do paciente contribuir no seu tratamento e evitar o acontecimento de eventos adversos.

### Limitações do estudo

Uma das limitações foi a pandemia da COVID - 19 (Coronavírus). que retardou a aplicação da cartilha aos pacientes do estudo. com maior risco de contaminação dos pesquisadores e pacientes. sendo mantidas as medidas sanitárias vigentes.

### Contribuições para a área da enfermagem

A enfermagem tem papel fundamental na assistência já que tem proximidade constante com o paciente. O cuidado precisa ser sistematizado e condicionado entre a equipe de saúde e do usuário para garantir a qualidade da assistência^(28)^.

## CONSIDERAÇÕES FINAIS

Acredita-se que esta tecnologia educativa possa servir como apoio essencial à equipe de saúde. Os profissionais têm a possibilidade de trabalhar o posicionamento do paciente como participante ativo de sua própria segurança. visto que essa nova condição facilita a assistência e estreita relação entre usuário e profissional.

A utilização dessa cartilha poderá atingir com mais assertividade uma cultura de segurança organizacional. uma vez que engloba a participação ativa do paciente. Percebe-se. porém. ainda. uma resistência na inserção de ferramentas tal qual fora apresentada. sem ter seu potencial totalmente utilizado. Para isso. a persistência na utilização do material pode contribuir para um cuidado mais seguro. um paciente e familiar bem informado e uma instituição com destaque no aspecto da Segurança do Paciente.
